# Effects of Storage Time and Temperature on Lipid Oxidation and Protein Co-Oxidation of Low-Moisture Shredded Meat Products

**DOI:** 10.3390/antiox8100486

**Published:** 2019-10-16

**Authors:** Hazrati Wazir, Shyan Yea Chay, Mohammad Zarei, Farah Salina Hussin, Nor Afizah Mustapha, Wan Zunairah Wan Ibadullah, Nazamid Saari

**Affiliations:** 1 Department of Food Science, Faculty of Food Science and Technology, Universiti Putra Malaysia, Serdang 43400, Selangor, Malaysia; hazratiwazir@gmail.com (H.W.); da_charming_me@hotmail.com (S.Y.C.); farahsalina@unikl.edu.my (F.S.H.); 2 Panel of Food Technology, Department of Technology and Natural Resources, Faculty of Applied Sciences and Technology, Universiti Tun Hussein Onn Malaysia, Pagoh Education Hub, KM 1, Jalan Panchor, Muar 84600, Johor, Malaysia; 3 Department of Food Science and Technology, School of Industrial Technology, Faculty of Applied Sciences, Universiti Teknologi MARA, Shah Alam 40450, Selangor, Malaysia; mzarei.mail@gmail.com; 4 Department of Food Technology, Faculty of Food Science and Technology, Universiti Putra Malaysia, Serdang 43400, Selangor, Malaysia; nor_afizah@upm.edu.my

**Keywords:** lipid oxidation, low moisture food, protein co-oxidation, ready-to-eat, shredded meat, *serunding*

## Abstract

Studies on the oxidative changes in meat-based, low-moisture, ready to eat foods are complicated due to complex food system and slow lipid-protein oxidative deterioration. The current study evaluates the oxidative changes over six months of storage on shredded beef and chicken products (locally known as *serunding*) for physicochemical analysis, lipid oxidation (conjugated dienes and malondialdehydes) and protein co-oxidation (soluble protein content, amino acid composition, protein carbonyl, tryptophan loss and Schiff base fluorescence) at 25 °C, 40 °C and 60 °C. The lipid stability of chicken *serunding* was significantly lower than beef *serunding*, illustrated by higher conjugated dienes content and higher rate of malondialdehyde formation during storage. In terms of protein co-oxidation, chicken *serunding* with higher polyunsaturated fatty acids (PUFA) experienced more severe oxidation, as seen from lower protein solubility, higher protein carbonyl and Schiff base formation compared to beef *serunding*. To conclude, chicken *serunding* demonstrates lower lipid and protein stability and exhibits higher rate of lipid oxidation and protein co-oxidation than beef *serunding*. These findings provide insights on the progression of lipid oxidation and protein co-oxidation in cooked, shredded meat products and could be extrapolated to minimize possible adverse effects arising from lipid oxidation and protein co-oxidation, on the quality of low-moisture, high-lipid, high-protein foods.

## 1. Introduction

Low-moisture, ready-to-eat (RTE) meat-based food refers to an animal-derived, cooked and processed product that is typically rich in protein and lipid, and is stabilised by a lowered water activity (*a*_w_ < 0.9) to inhibit bacterial growth [1,2,3]. The consumption of meat-based RTE foods has increased, mainly because of a modern and busy lifestyle, which demands for convenience and minimal preparation time [1]. Moreover, its advantages of being readily packed, does not require additional cooking to be safely consumed, energy dense, light-weight (moisture removed) and good stability at varying environments for an extended period of time make it a popular choice among travellers [4].

Despite the advancement in packaging technology, lipid oxidation and subsequent co-oxidation with non-lipid molecules (particularly protein) remains inevitable and represents the most important mechanism of quality degradation in meat-based RTE products [5]. Protein co-oxidation is strongly interlinked with lipid oxidation in meat-based RTE products because lipid and protein are closely associated in food structures and in membranes. Indeed, most intermediate and final products from lipid oxidation would react with protein [6,7]. The comprehensive mechanisms involved in protein co-oxidation with lipid were elaborated by Schaich [7].

Studies on the protein co-oxidation with lipid in meat-based RTE products is complicated because the progress of lipid-protein co-oxidative degradation in low moisture foods has been relatively slow (which takes months to years) compared to high moisture foods [6,8,9]. The current study evaluates the long-term storage effect (six-months) at different temperatures (25 °C, 40 °C and 60 °C) on lipid and protein co-oxidation in cooked, shredded RTE meat products (*serunding*), as it represents a complex food model of high lipid, high protein and low moisture. Additionally known as meat floss or desiccated meat, *serunding* is a popular, traditional local cuisine that is uniquely flavoured with coconut milk. The use of dried, cooked meat and coconut milk as the major ingredients causes the final product to be low in moisture but rich in lipid and protein. The significance of the current work in relation to food safety is seen in the health concerns caused by consuming food products that have undergone lipid oxidation and protein co-oxidation. Estévez and Xiong [10] demonstrated that, health risks such as oxidative stress and related diseases, are associated with the intake of oxidised protein. Thus, evaluating the oxidation and co-oxidation processes in food products would serve as a basis for future studies related to food-body cell interactions.

To the authors’ best knowledge, the effect of long-term storage on lipid oxidation and protein co-oxidation (occurs after lipid oxidation, through pathways with highly reactive, oxidised lipid products) in shredded meats has not been reported elsewhere. Using two shredded meat products from beef and chicken, the present study evaluates the changes in physicochemical properties (fatty acid composition, *a*_w_, colour), primary (conjugated dienes) and secondary lipid oxidation products (malondialdehydes) on a weekly/biweekly basis. Protein damage in these products, as a result of lipid-protein co-oxidation, is also evaluated using several protein co-oxidation markers (protein solubility, protein carbonyl content, tryptophan loss and Schiff base formation). However, the current work presents some limitations such as lacking in the study that adds antioxidants into RTE meat products to evaluate the oxidative processes during the lag phase, as well as detailed carbonyl measurements. In the future, the detection of specific protein carbonyl products, including α-aminoadipic semialdehydes and γ-glutamic semialdehydes, could be accommodated following the work by Villaverde and Estévez [11].

## 2. Materials and methods

### 2.1. Materials

Fresh beef and chicken breast meats were purchased from a local market in Kelantan, Malaysia. Meats were trimmed off visible fat and immediately kept at 4 °C prior to the making of shredded meat products. All chemicals used were of high performance liquid chromatography (HPLC) or analytical grade and were obtained from Acros organics (Geel, Belgium) and Sigma-Aldrich (St Louis, MO, USA). Purified water used for cooking was obtained by passage through a Milli-Q system (Millipore Corp., Bedford, MA, USA).

### 2.2. Preparation of Shredded Meat Products

Beef and chicken meats were sliced into thick pieces of about 5 × 5 × 10 cm in size, then boiled overnight in water (meat:water ratio at 1:1.5, *w*/*v*) until tender, drained, and allowed to cool. The boiling water was added with few slices of tamarind to remove the raw smell of meat. The remaining water was collected as a meat broth. When adequately cooled, the fibres of cooked meat were manually shredded to obtain strands of meat. The sauce was prepared using the following ingredients: Coconut milk (25.42 g/100 g), sugar (6.78 g/100 g), onion (22.03 g/100 g), garlic (3.39 g/100 g), ginger (3.39 g/100 g), salt (1.69 g/100 g), tamarind paste (*Garnicia atroviridis*) (0.03 g/100 g), dried chilli (1.69 g/100 g) and freshly grounded coriander seeds (1.69 g/100 g). All ingredients were blended using the meat broth until a homogenous mixture was formed. The mixture was then concentrated in a saucepan by heating at 80 °C–90 °C with continuous stirring to prevent charring. When concentrated to 50% of its original volume, shredded meats were added (33.89 g/100 g) and stirred continuously until the desired dryness was achieved. The whole cooking process took up to 8 h. Upon cooling, shredded beef and chicken meats were sealed into light-proof, gas-impermeable individual packagings. During six-months storage, shredded meat samples were arranged in single layers on well-ventilated trays and incubated at 20 °C, 40 °C and 60 °C, respectively. For control, samples were stored at −80 °C to prevent oxidation reaction. 

### 2.3. Physicochemical Quality Assessment on Shredded Meat Products

#### 2.3.1. Proximate Composition

The proximate analysis (moisture, ash, crude fat and crude protein) was performed according to the Association of Official Analytical Chemists (AOAC) official method [12]. All determinations were done in triplicates. 

#### 2.3.2. Reducing Sugar Content

The reducing sugar analysis was done following the method described by Utrera et al. [13] with slight modification. Reducing sugar was first extracted from shredded meats using distilled water at 1:4 (*w*/*v*) ratio by a homogeniser (IKA Ultra-Turrax, Staufen, Germany). The mixture was homogenised at 1600 rpm and 25 °C for 2 min. A homogenated sample was centrifuged for 3 min at 800 × *g*, then filtered and made up to 25 mL using distilled water. Extract (0.5 mL) was added into 0.5 mL of 1% 3,5-dinitrosalicylic acid (DNS) solution and placed in a boiling water bath (100 °C) for 5 min. After cooling, the reaction mixture was further diluted before the absorbance was measured at 540 nm. Reducing the sugar content was calculated from a glucose standard curve (R^2^ = 0.9990, 0.0–1.0 mg/mL). Results were expressed as mg glucose/g sample.

#### 2.3.3. Fatty Acid Composition

Fatty acid methyl esters (FAMEs) were prepared by trans-esterification of the oil using a sodium methoxide complex as catalyst, with slight modification from The American Oil Chemists’ Society method Ce 1-62 [14]. The analysis of FAME was conducted using a gas chromatography system (Agilent 6890N Network GC System), fitted with a flame ionisation detector and an automated liquid sampler (Agilent 7683 series). The entire system is controlled by the Chemstation® Software. FAMEs were separated on a DB-WAX capillary column (30 m × 0.25 mm ID, 0.25 μm film thickness). All instruments were supplied by Agilent Technologies Inc., Santa Clara, CA, USA. The column was initially set at 100 °C for 2 min, before being increased to 230 °C at 5 °C/min and lastly held for 10 min at 230 °C. The carrier gas was helium (flow rate = 1.0 mL/min) controlled at 103.4 kPa. The sample volume of 1 μL was injected with a split ratio of 1:20. Peak identification was done by comparison to FAMEs standard (Supelco Park, Bellefonte, PA, USA). The fatty acid profile was expressed in % using the area normalisation method, based on the following equation:

(1) $$\frac{Individual\;FAME\;area}{Total\;area}\times 100\%$$

#### 2.3.4. Water Activity, *a*_w_

Water activity (*a*_w_) was determined by inserting a plastic disposable cup filled with 5 g–6 g of sample into the sample drawer of the *a*_w_ meter (AquaLab, Decagon Devices, Inc., Pullman, WA, USA). The sample was levelled prior to determination. 

#### 2.3.5. Colour Measurement

Colour measurement was performed on the sample surface using a Minolta Chromameter (Minolta Camera Corp., Meter Division, Ramsey, NJ, USA), which consisted of a measuring head (model CR-300), with an 8 mm diameter measuring area and a data processor (model DP-301). Measurements were made at room temperature with an illuminant D65 and a 0° angle observer. L*, a* and b* values (CIE L*a*b* colour system) were assessed as a measure of lightness, redness and yellowness, correspondingly.

### 2.4. Lipid Oxidation Analysis

#### 2.4.1. Lipid Extraction and Total Lipid Content

Total lipid was extracted in accordance with the procedure detailed by Ibadullah [15]. Shredded meat samples were mixed with chloroform: Methanol (2:1, *v*/*v*) in 1:5 (*w*/*v*) ratio, flushed with argon and allowed to stand for 30 min, then centrifuged at 14,000 × *g*, 4 °C for 30 min. The extraction steps were repeated once and both supernatants were combined. The pooled supernatant was evaporated to determine the amount of lipid, expressed as a percentage of lipid recovery (%) based on the initial sample weight. Dried lipid extracts were flushed with argon, sealed, and stored at −20 °C until further analysis. The meal from *serunding* samples was dried for 2 h–3 h at room temperature to produce a fat-free sample for use in protein analysis.

#### 2.4.2. Conjugated Dienes (CD)

The CD content was determined by a modification from The American Oil Chemists’ Society standard method Th 1a-64 [16]. Briefly, 30 µL of lipid extract, obtained from Section 2.4.1, was added into 10 mL of isooctane and the absorbance was measured at 234 nm against isooctane (blank). Concentrations of CD (mM) were calculated from Beer’s Law using a molar extinction coefficient of 29,500 (L mol^−1^ cm^−1^) for isooctane. CD was expressed as mmol CD/mol lipid. 

#### 2.4.3. Thiobarbituric Acid Reactive Substances (TBARS) Value

The TBARS concentration was determined using the Food TBARS Assay Kit (Oxford Biomedical Research Inc., Rochester Hills, MI, USA). Shredded meat samples were mixed with distilled water at 1:2 (*w*/*v*) ratio, then homogenised at 1000 rpm for 3 min to form a smooth suspension. The TBA reagent (consisting of 2.5 g of 2-thiobarbituric acid + 50 mL of proprietary acid catalyst) was added to the suspension at 1:1 (*v*/*v*) ratio. The mixture was then vigorously agitated for 1 min using the vortex mixer to produce a homogenised sample. The reaction was allowed for 60 min at room temperature. Quantification was performed by measuring the absorbance at 532 nm. TBARS concentrations were determined using a malondialdehyde (MDA) standard calibration curve (R^2^ = 0.9999, 0 to 3.0 mg/L). The results were expressed as mg MDA equivalents/kg sample.

### 2.5. Protein Co-oxidation Analysis

#### 2.5.1. Soluble Protein Content

Briefly, de-oiled samples from Section 2.4.1 were extracted overnight using a sodium phosphate buffer (0.2 M, pH 7.9) containing 0.02% sodium azide with constant shaking at 150 rpm. The mixture was then centrifuged at 14,000 × *g* for 30 min. The supernatant was collected, flushed with argon gas and stored at −80 °C until analysis. The soluble protein content was determined using the method from Bradford [17] and expressed in percentage as the total soluble proteins per sample weight (*w*/*w*, dry basis). Bovine serum albumin (BSA) was used as the protein standard. 

#### 2.5.2. Amino Acid Composition

The amino acid composition was determined using the PicoTag pre-column phenyl-isothiocyanate (PITC) derivatisation method with slight modification [18]. The HPLC technique was applied using a C18 reversed phase column (Hypersil GOLD^TM^, Thermo Scientific, 250 mm × 4.6 mm ID, 5 μm particle size). The mobile phases consisted of buffer A (0.1 M ammonium acetate, pH 6.5) and buffer B (0.1 M ammonium acetate + acetonitrile + methanol, 44:46:10 *v*/*v*/*v*, pH = 6.5). the column temperature was maintained at 43 °C in a gradient run of buffer A (100–0% in 50 min) and buffer B (0–100% in 50 min) at a flow rate of 1 mL/min. Absorbance was recorded at 254 nm. Amino acids were identified and quantified by comparing their peaks with that from external standards. Determinations were conducted in triplicates and results were reported as mg amino acid/g sample. 

#### 2.5.3. Protein Carbonyl

Carbonyl content was determined by derivatisation with 2,4-dinitrophenyl hydrazine (DNPH) as described by Soglia, Petracci and Ertbjerg [19] with some modifications. The shredded meat sample was added into a phosphate buffer (20 mM, pH 6.5 containing 0.6M NaCl) at a ratio of 1:10 (*w*/*v*). Four aliquots (0.2 mL each) were treated with 1.0 mL of ice-cold 10% trichloroacetic acid (TCA) to precipitate the proteins. After centrifugation at 4500 × *g* for 3 min, the supernatant was discarded. Two aliquots were treated with 0.5 mL of 10 mM DNPH dissolved in 2.0 M hydrochloric acid (HCl) while the other two remaining aliquots were treated with 0.5 mL of 2.0 M HCl (blank). After 1 h of reaction at room temperature, 0.5 mL of ice-cold 20% TCA was added. Samples were then centrifuged and the supernatant was discarded. Excess DNPH was removed by washing three times with 1 mL of ethanol:ethylacetate (1:1, *v*/*v*). The pellets were dissolved in 1 mL of 6.0 M guanidine hydrochloride in 20 mM phosphate buffer (pH = 6.5). Absorbance was read against blank at 370 nm to estimate the carbonyl concentration (represented as protein hydrazones). Another set of absorbance was measured at 280 nm to estimate the protein concentration. Carbonyl concentration was calculated based on the molar absorptivity for protein hydrazones (22,000 M^−1^ cm^−1^) and correction factor for hydrazone peak tail overlapping (0.43) using the following equation [20,21]:

(2) $$\frac{A_{370}\mathrm{sample}-A_{370}\mathrm{blank}}{22,000\times \left[A_{280}\mathrm{sample}-\left(A_{370}\mathrm{sample}-A_{370}\;\mathrm{blank}\right)\times 0.43\right]}\times 10^{6}$$

Carbonyl concentration was expressed as nmol DNPH/mg protein.

#### 2.5.4. Tryptophan Loss and Schiff Base Fluorescence Spectroscopy

Protein co-oxidation was monitored using a spectrofluorometric assay according to the method described by Ibadullah [15]. An argon-flushed supernatant from Section 2.5.1 was used. Intrinsic fluorescence from aromatic amino acids (primarily tryptophan) was determined by recording fluorescence emission spectra (λ_em_) from 300 nm to 450 nm with excitation at 280 nm, using 9 nm bandwidth and excitation/emission slits at 10 nm. Emission intensity of tryptophan was recorded at 330 nm. Presumptive formation of Schiff base complexes between lipid carbonyls and protein amino groups were detected by recording λ_em_ from 400 nm to 650 nm with excitation at 350 nm. Emission intensity was recorded at 430 nm. All spectra were recorded in synchronous mode that produced both excitation and emission curves, displayed as an average of the three spectra for each sample. 

### 2.6. Statistical Analysis

All results were evaluated using one-way analysis of variance (ANOVA). The comparison of lipid and protein co-oxidation markers between the shredded chicken and beef was measured using a general linear model. The mean difference between groups were assessed using Tukey’s post hoc test operating at a 5% level of significance (*p* < 0.05). All statistical analysis was performed using the Minitab 16 statistical software (Minitab Inc., State College, PA, USA).

## 3. Results and Discussions

### 3.1. Physicochemical Analysis of Shredded Meat Products

#### 3.1.1. Proximate Composition and *a*_w_

Table 1 depicts the proximate analysis for two shredded meat products, namely beef and chicken. The low moisture content (<10%) and *a*_w_ (<0.85) in these products allowed them to be classified as low-moisture foods [22] with an extended shelf life compared to fresh meat products. While beef *serunding* demonstrated protein and fat contents that were comparable to literature, chicken *serunding* showed lower protein and higher fat than those reported. However, the fat content in chicken *serunding* remained similar to that from beef, indicative of oil absorption from coconut milk that occurred at a similar rate between both meat products under the same processing conditions. The lipid-rich coconut milk was fully absorbed into the meat fibre during long cooking hours, thus explained the high fat content detected in the final product. High fat content then leads to a lowered protein content in both samples in the present study, as the proximate analysis is reported on “per 100%” basis. A higher fat proportion in 100 g of sample indicates a lower protein proportion in the same 100 g.

In dried food products, water activity (*a*_w_) is a critical parameter which affects the stability and shelf life. Numerous deteriorative reactions, including lipid oxidation, Maillard browning, and enzymatic reactions still occur at relatively low *a*_w_ values [26]. Specifically, for oxidative deterioration, Sun, Senecal, Chinachoti and Faustman [26] demonstrated that lipid oxidation was favoured at low *a*_w_ (0.0–0.33) in freeze-dried beef patties stored at 49 °C while Cheng et al. [27] showed that lipid oxidation still occurred at *a*_w_ = 0.33 in formulated infant milk powder. Thus, the measurement of *a*_w_ is crucial to determine the stability for dried foods stored over long time. It was found that, after six months of storage, the *a*_w_ for the control sample stored at −80 °C increased significantly from 0.381 to 0.403 for beef *serunding* and from 0.431 to 0.463 for chicken *serunding*. This shows that the increment in *a*_w_ is inevitable even at an ultra-low storage temperature. For samples stored at 25 °C, 40 °C and 60 °C, *a*_w_ increased more rapidly at higher temperature than at lower temperature when compared across week zero to week 24 (data not shown). This shows that temperature plays a critical role in determining the quality of dried food products over long storage, whereby higher temperatures will cause a more rapid increment in *a*_w_ thus adversely affecting the product quality.

#### 3.1.2. Fatty Acid Composition (FAC)

The FAC in dried, shredded meat products is more critical than its total fat content because the vulnerability towards lipid oxidation depends largely on the fatty acid (FA) unsaturation degree. To date, no data is available on the FAC of *serunding*. Table 2 tabulates the FAC of beef and chicken *serunding* in comparison with raw and cooked beef and chicken. It is noticed that both *serunding* products showed significantly higher saturated fat than raw and cooked meats, due to the addition of coconut milk that is rich in saturated fat. Although the usage of coconut milk causes an increment in the saturated fat content, coconut milk is known to contain mainly medium chain triglycerides (MCT, 6–12 carbon chain length) that provides much health benefits to the body, such as weight reduction, increased insulin sensitivity and raise “good cholesterol” level [28,29,30]. The total MCT contents in shredded meat products were fairly high, recorded 56.02% in beef and 63.51% in chicken, respectively.

Another noteworthy observation is the significant reduction in unsaturated FA content (both mono- and polyunsaturated) in *serunding* compared to their respective raw and cooked form. Beef *serunding* contained only 7.29% unsaturated FA while raw and cooked beef contained much higher amounts at 51.35% and 56.26%, respectively. Chicken *serunding* contained only 10.21% unsaturated FA while raw and cooked chicken contained 62.18% and 66.29%, respectively. This reduction in shredded meat products is due to long hour cooking which decreases the FA unsaturation degree. Moreno et al. [33] showed in their study that, when different edible oil was heated, a significant drop in the unsaturation percentage was detected, as a result of spontaneous thermal oxidation which deteriorated polyunsaturated fatty acids (PUFA) into hydroperoxides. The long heating process thus explained the reduced FA unsaturation degree and low monounsaturated fatty acids (MUFA) and PUFA content in shredded meats. The stability of unsaturated FA is improved by the addition of several functional ingredients such as onion, garlic, chilli, ginger, tamarind and coriander seeds which act as natural antioxidants. Each ingredient has certain antioxidative components, such as capsaicin in red chilli, zingeron in ginger, alin and alisin in onion and garlic [34]. These natural antioxidants prevent the complete oxidation of MUFA and PUFA, leaving behind a minor amount that remained intact and was detected in the final products.

#### 3.1.3. Colour 

**Figure 1:**
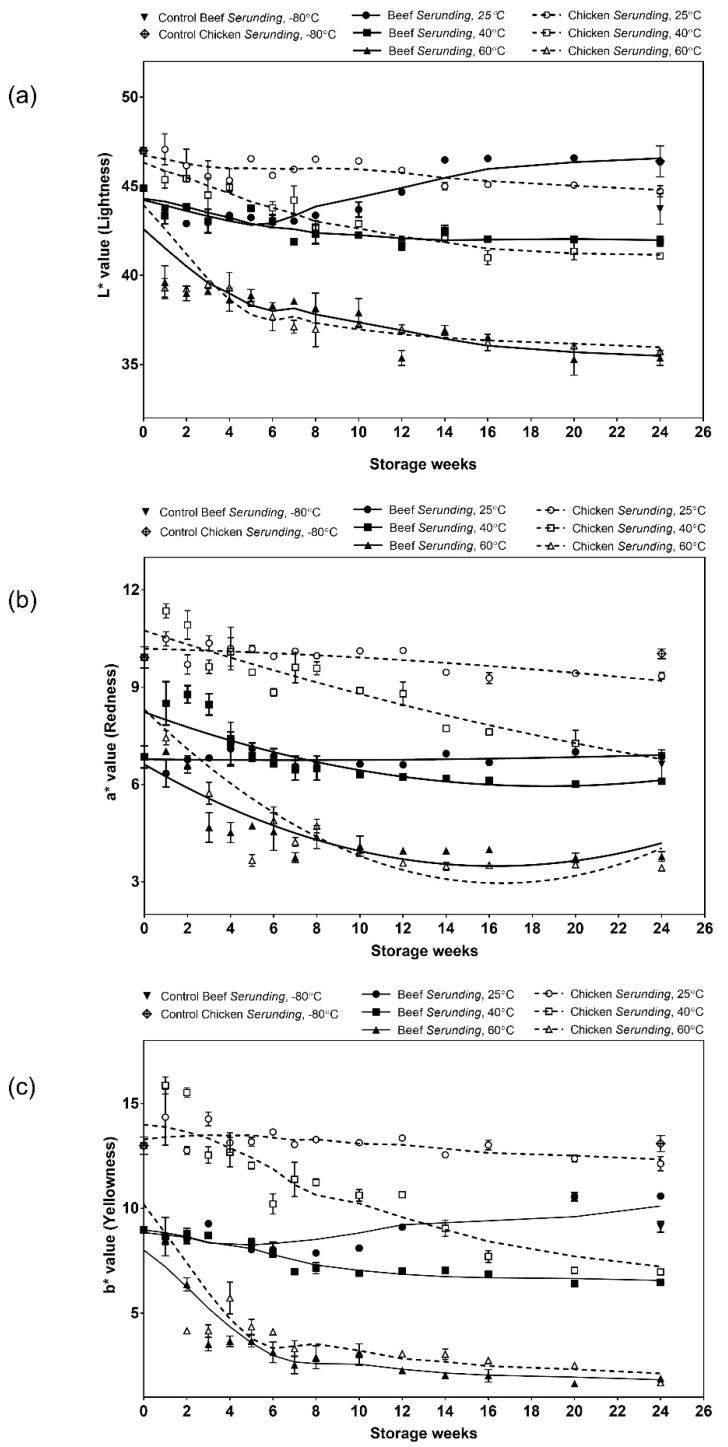
Colour measurements of (**a**) lightness; (**b**) redness and (**c**) yellowness on *serunding* products during six-months storage.

*Serunding* appears brown in colour. This browning is due to several factors: (i) Denaturation of myoglobin, (ii) Maillard reaction and (iii) aldol condensation between lipid carbonyl and protein. As a meat-based food, the myoglobin in *serunding* mainly exists in a denatured form after cooking. Denaturation of myoglobin eventually results in brown colour formation (the cooked meat colour) [35]. Maillard reaction is mainly responsible for browning during a long cooking process (8 h). It takes place between reducing sugar and amino-bearing compounds (meat protein) and is accelerated by heat. From Table 1, both beef and chicken *serunding* contains reducing sugar at 1.09 mg and 1.02 mg glucose/g sample, respectively. These sugars reacted with protein molecules when heat was applied during the cooking process, intensifying the formation of brown pigments and contributed towards the final brown colour in the product. During 24 weeks of storage, aldol condensation took over progressively when lipid carbonyl was formed via spontaneous oxidation. These lipid carbonyls would substitute reducing sugar as a carbonyl source to further react with protein and enhance darkening. This was explained by the significant decrement in L*, a*, b* values over 24 weeks of storage for samples stored at 40 °C and 60 °C (Figure 1), indicative of product darkening over time. The colour changes in samples stored at lower temperature (25 °C) were insignificant over 24 weeks of storage. This suggested that a heat-driven Maillard reaction coupled with an aldol condensation and caused enhanced darkening in samples stored at higher temperatures of 40 °C and 60 °C, compared to that stored at 25 °C. 

Another noteworthy observation was on the samples stored at 60 °C. At this temperature, both beef and chicken *serunding* showed a faster rate of product darkening compared to 25 °C and 40 °C starting from week two onwards. Additionally, the packaging for both samples became bloated and the samples turned greasy and brittle, to the point that it was easily crumbled into ashy pieces when touched with fingers. These observations concluded that the storage of low-moisture, high-lipid, high-protein food product at an accelerated temperature (60 °C) was not feasible. However, the lipid oxidation and protein co-oxidation processes at an accelerated temperature is worth being studied and is thus reported in the following section.

### 3.2. Lipid Oxidation

#### 3.2.1. Extracted Lipid

**Figure 2:**
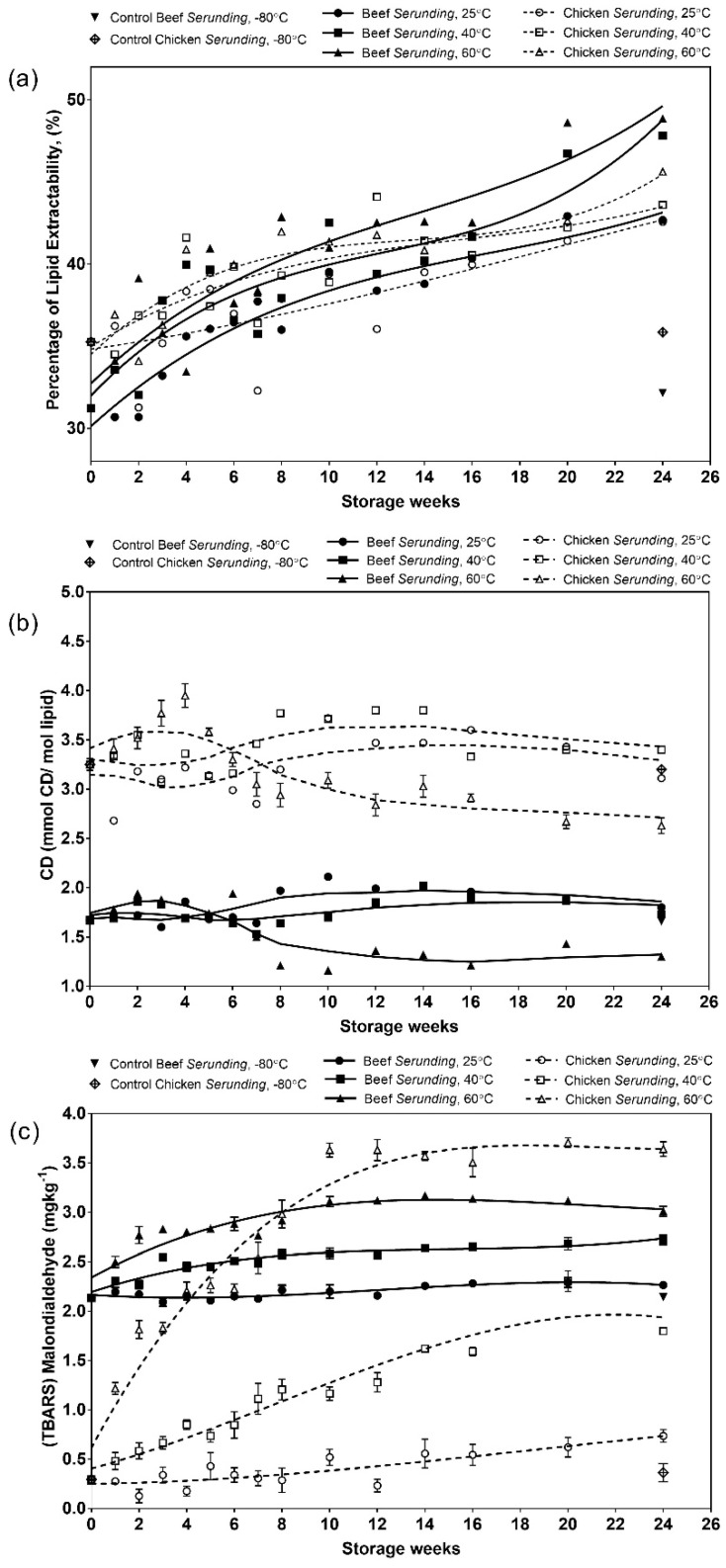
Lipid oxidation markers in terms of (**a**) lipid extractability; (**b**) conjugated dienes (CD) and (**c**) malondialdehydes for beef and chicken *serunding* at different storage temperatures over 24 weeks.

Based on Figure 2a, there is a gradual increase (*p* < 0.05) in lipid extractability over incubation time at all storage temperatures. The increased lipid extractability may be due to advanced disruption in the meat structures at the later stage of storage which caused oil separation from *serunding* samples and increased the accessibility to the solvent. It was observed that oily layers built up in the packaging for *serunding* samples at 60 °C from week four onwards. Due to severe damage of the meat structure, the melted fat crystals and the entrapped oil can no longer be held within the food matrices [15] and was thus easily extracted.

#### 3.2.2. Conjugated Dienes (CD)

CD is a primary lipid oxidation product that is formed when the double bonds in unsaturated fatty acids are shifted, converting nonconjugated fatty acids into conjugated ones. From Figure 2b, the CD contents for chicken *serunding* are consistently higher than that for beef *serunding* irrespective of storage weeks and temperatures. This is due to the presence of higher amounts of PUFA in chicken meat than in beef, making chicken *serunding* a more favourable substrate to initiate lipid oxidation. Another observation is that the CD contents in samples stored at 25 °C and 40 °C peaked at a later stage (week 10–12). This is in contrast with the CD content for samples stored at 60 °C, which peaked much earlier at week four, followed by a continuous decrement until week 24. This indicates the adverse effect from a high temperature that rapidly speeds up the primary lipid oxidation process and jeopardizes the quality of the final product, further justifying the unsuitability of storing low moisture, high-lipid, high-protein food products at 60 °C. As discussed earlier in Section 3.1.2, the addition of several ingredients which act as natural antioxidants help in delaying CD formation at 25 °C and 40 °C, as antioxidants remain comparatively stable at these temperatures than storage at the highest temperature (60 °C). The antioxidative potential of natural antioxidants to suppress lipid oxidation had been elucidated in cooked chicken products by CD measurements [36,37]. This proves the capacity of natural antioxidants to reduce CD formation in meat products and explains its role in the delay of CD formation in shredded meats. The decrement in CD levels during the later stage of storage could be explained by the formation of secondary lipid oxidation products of lower molecular weight, e.g., aldehydes and ketones [38].

#### 3.2.3. Malondialdehydes (MDA)

MDA is a common marker product for secondary lipid oxidation, particularly in meat muscle foods [6]. It is typically assessed as thiobarbituric acid reactive substances (TBARS). From Figure 2c, the MDA level shows a consistent increment from week zero to week 24 in all samples, with chicken *serunding* exhibiting a higher MDA formation rate than beef in the following order: 60 °C > 40 °C > 25 °C. The early formation of MDA, particularly in chicken *serunding*, is unexpected because in typical oxidation pathways in a low moisture food system, the formation of secondary oxidation products should occur at a later stage of storage after the formation of CD has ceased. This opposite trend of prominent MDA formation in chicken *serunding* at early storage is due to its higher content of polyunsaturated fats and phospholipids, which have higher susceptibility towards oxidative reaction and is responsible for rapid secondary lipid degradation, as detected in the high MDA content during early storage. Towards the end of storage (week 16 onwards), the MDA formation reached a stagnant phase regardless of sample types and temperatures. This phenomenon suggested the occurrence of other reactions such as the active radical transfer with non-lipid molecules (protein) that took place parallelly with the MDA formation. MDA (α,β-unsaturated aldehydes) is an electrophile that can react with nucleophilic groups in protein. Previous studies showed that MDA could covalently bind with lysine residues to form a MDA-lysine adduct [39], react with N-terminus of peptides and electrophilic ε-amino group of glutamine to form Schiff base adducts and also bind with cysteine side chain due to the presence of the thiol group [40]. These possible pathways of the MDA interaction with protein may explain the stagnant MDA formation after 16 weeks of storage. Based on the results from CD and MDA, chicken *serunding* showed higher lipid oxidation and lower lipid stability than beef *serunding* (*p* < 0.05) throughout 24 weeks of storage.

### 3.3. Protein Co-oxidation

In the present study, the susceptibility of meat *serunding* towards protein co-oxidation was assessed by various measurements including protein solubility, depletion of protein components (amino acid composition and tryptophan loss) and the formation of protein co-oxidation products (carbonyls and Schiff bases). Primary and secondary lipid oxidation products could act as substrates to initiate protein co-oxidation. Thus, once lipid oxidation occurs, protein co-oxidation then takes place subsequently. These reactions cause variations in the amino acid composition, which leads to decreased solubility, tryptophan losses and formation of carbonyl compounds.

#### 3.3.1. Soluble Protein Content

**Figure 3:**
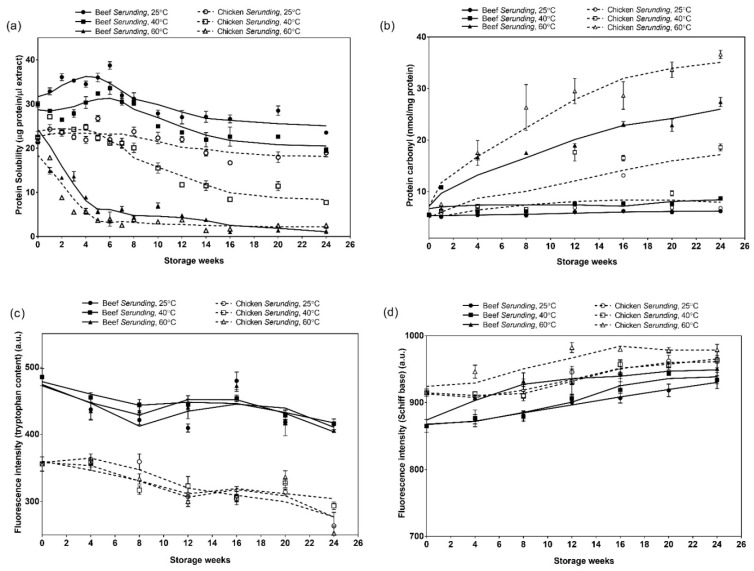
Protein co-oxidation markers in terms of (**a**) protein solubility; (**b**) protein carbonyl; (**c**) tryptophan content and (**d**) Schiff base fluorescence for beef and chicken *serunding* at different storage temperatures over 24 weeks.

Figure 3a shows the protein solubility of both beef and chicken *serunding* at 25 °C, 40 °C and 60 °C over 24 weeks of storage. At higher storage temperature, protein solubility decreased more rapidly in both *serunding* products, of which samples at 60 °C showed a significant drop starting from as early as week two while samples at 25 °C and 40 °C showed a drop only from week 10 onwards. Additionally, chicken *serunding* demonstrated lower protein solubility as compared to beef *serunding* at all storage temperatures. The changes in protein solubility were parallel with the occurrence of lipid oxidation that is more rapid in chicken *serunding* than that in beef *serunding*. These observations suggested that lipid oxidation products could react with protein molecules to cause modifications that reduce protein solubility.

#### 3.3.2. Amino Acid Composition

Table 3 tabulates the amount of amino acid residues for beef and chicken *serunding* before and after 24 weeks of storage. Most amino acids recorded a significant reduction after 24 weeks of storage, indicating that protein molecules in both *serunding* products experienced structural alteration during storage. Amino acid residues, particularly lysine, arginine, histidine and proline, are prone to oxidise into carbonyls that can adversely affect the functionality of meat proteins in cooked meat products [41]. This is because these amino acids are located primarily on protein surfaces and have readily abstractable hydrogens, thus they become the primary targets for oxidizing lipids to attack and initiate protein co-oxidation [15,42]. A decrease in these amino acids in both *serunding* products partially explained the formation of protein carbonyl as discussed in the following section. On the other hand, most of the hydrophobic amino acids, such as glycine, alanine and isoleucine, showed a significant loss in both *serunding* during storage. Initially, these amino acids have no promptly abstractable hydrogens and are minimally involved in hydrogen bonding and embedded within the insides of protein molecules. Upon heat treatment during cooking of *serunding*, the proteins were denatured and unfolded, exposing these amino acids to the attack by lipid radicals that were produced progressively over time [6,15], thus reducing the amino acid content as detected at the end of storage.

#### 3.3.3. Protein Carbonyl

The extent of protein co-oxidation in *serunding* samples were tracked further by evaluating the most significant index in protein co-oxidation, i.e., formation of carbonyl compounds. The quantitation of carbonyl compounds spectrophotometrically using 2,4-dinitrophenylhydrazine is a widely used method for evaluating protein co-oxidation in muscle foods. Previous studies have discussed the success quantification of protein carbonyl in a large range of meat samples including beef, chicken and porcine muscles [43,44].

Figure 3b shows that the formation of protein carbonyl takes place in a temperature-dependent manner. At the highest temperature of 60 °C, protein carbonyl formation is the most prominent, followed by 40 °C and lastly 25 °C. This observation fosters the idea that the storage of low moisture foods at an accelerated temperature is not feasible. High levels of protein carbonyl at the early stage of 60 °C storage is explained by several pathways which cause the accumulation of carbonyls, including protein interaction with secondary lipid oxidation products (MDA), fragmentation of protein backbones through the *α*-amidation pathway and *β*-scission as well as direct oxidation of amino acid side chains including arginine, lysine, proline and threonine [43]. When comparing at the same temperature, chicken *serunding* showed a higher carbonyl formation rate than beef *serunding*. This finding was in line with the higher lipid oxidation rate and lower protein solubility in chicken *serunding* as discussed earlier, which makes it more susceptible towards protein co-oxidation.

#### 3.3.4. Tryptophan Loss and Schiff Base Fluorescence Spectroscopy

Tryptophan residue, an aromatic amino acid, is known for being preferential targets of lipid oxidative products, particularly MDA. Tryptophan is located primarily on protein surfaces where it is in closest contact with oxidizing lipids and therefore, vulnerable during oxidative reactions [42,45,46]. These reactions cause conformational changes in protein molecules and lead to a decrease in the intrinsic fluorescence of tryptophan. Thus, the loss of tryptophan is used as one of the markers to evaluate protein co-oxidation in meat. In the present study, the loss of tryptophan is depicted in Figure 3c. The tryptophan fluorescence emission in chicken *serunding* was constantly lower than beef *serunding* at all weeks, indicating the lower amount of tryptophan in chicken than in beef. In addition, chicken *serunding* depicted a higher tryptophan loss (−26% at 25 °C, −18% at 40 °C and −29% at 60 °C) than beef *serunding* (−14% at 25 °C, −14% at 40 °C and −16% at 60 °C) compared to the control after 24 weeks of storage. The lowered tryptophan content and higher tryptophan loss in chicken *serunding* could be explained by the higher rate of MDA formation in chicken *serunding* as shown in Figure 2c. The higher amount of MDA in chicken *serunding* would actively attack tryptophan molecules and disrupt its native structure, therefore lowering the respective fluorescence emission.

The Schiff base formation involves the production of stable radicals primarily from the reaction between lysine, histidine, glutamine or cysteine with reactive lipid oxidative products, which fluoresce at the conjugated structure –N=CH-CH=CH- [15]. Specifically, it involves the reaction between an electrophilic group at the carbonyl structure of aldehydes from secondary lipid oxidative products (MDA) and a nucleophilic group on proteins (electron-rich side chain of amino acids), to form Schiff base adducts [47], which act as an important indicator of protein co-oxidation. The Schiff base fluorescence emissions of *serunding* products are shown in Figure 3d. Chicken *serunding* exhibited higher Schiff base fluorescence compared to beef *serunding* at all temperatures. This is due to the lipid oxidation that is more active in chicken *serunding* to produce higher amounts of reactive radicals to take part in protein co-oxidation, releasing more Schiff base compounds. Secondly, the active involvement of lysine during the Schiff base formation explained the reduced lysine content in both *serunding* products, as depicted in Table 3. Lastly, the Schiff base was formed continuously even towards the end of the storage, signifying the progressive formation of protein co-oxidation products in *serunding* despite the fact that it is a low-moisture food with slower oxidation rate compared to intermediate or high-moisture foods.

## 4. Conclusions

Using shredded meat as a food model, the current study demonstrates the occurrence of lipid oxidation and protein co-oxidation in a low-moisture, high-lipid, high-protein food system. Storage temperature depicts a significant impact on the product quality, whereby an accelerated temperature (60 °C) causes major deteriorations in the final product and is thus not feasible for storage. Chicken *serunding* is more prone towards lipid oxidation and protein co-oxidation than beef, as seen in all oxidation marker analysis, due to a higher PUFA content in chicken meat. Higher PUFA also speeds up the occurrence of secondary lipid oxidation, demonstrated by the high built-up of the MDA content in chicken *serunding* during early storage. The rate of oxidation reactions for chicken *serunding* does not follow the same pattern, faster than beef *serunding.* These results can be taken into consideration when proposing effective processing/storage conditions to minimize possible adverse effects arising from lipid-protein co-oxidation, on the quality of cooked, shredded meat products.

## Figures and Tables

**Table 1:** Proximate analysis (%), *a_w_* and reducing sugar content (mg glucose/g serunding) for beef and chicken *serunding* obtained from present data and reported data.

Component.	Present Data		Reported Data^*^	
	Beef	Chicken	Beef	Chicken
Moisture	6.36 ± 0.03 ^a^	7.46 ± 0.07 ^b^	4.20–12.12	4.32–13.56
Protein	23.60 ± 0.02 ^a^	21.77 ± 0.12 ^b^	19.86–36.39	29.71–40.72
Fat	31.99 ± 0.10 ^a^	32.30 ± 0.36 ^a^	3.20–39.00	6.04–21.98
Ash	5.20 ± 0.01 ^a^	4.97 ± 0.09 ^b^	4.08–5.16	3.17–5.91
Carbohydrate (by difference)	32.85 ± 0.15 ^a^	33.50 ± 0.53 ^a^	N/A	N/A
Water activity Reducing sugar	0.381 ± 0.003 ^a^ 1.09 ± 0.027 ^a^	0.431 ± 0.002 ^b^ 1.02 ± 0.033 ^b^	0.410–0.640 N/A	N/A N/A

Means with different superscripts were significantly different at *p* < 0.05. * Reported data were obtained from three literatures [23,24,25].

**Table 2:** Fatty acid composition (%) of beef and chicken *serunding* compared with reported data on raw and cooked meats. Means with different superscripts were significantly different at *p* < 0.05. Reported data were obtained from two literatures [31,32].

	Present Data		Reported Data			
Sample	Beef *Serunding*	Chicken *Serunding*	Raw		Cooked	
			Beef	Chicken	Beef	Chicken
C6:0	0.67 ± 0.01 ^a^	0.73 ± 0.01 ^b^	-	-	-	-
C8:0	7.72 ± 0.10 ^a^	8.43 ± 0.08 ^b^	-	-	-	-
C10:0	5.85 ± 0.05 ^a^	6.50 ± 0.09 ^b^	-	-	-	-
C12:0	41.78 ± 0.33 ^a^	47.85 ± 0.70 ^b^	-	-	-	-
C14:0	15.51 ± 0.08 ^a^	17.29 ± 0.17 ^b^	-	-	-	-
C14:1	0.10 ± 0.01	-	-	-	-	-
C16:0	11.01 ± 0.10 ^b^	8.32 ± 0.37 ^a^	-	-	-	-
C16:1	0.45 ± 0.00 ^b^	0.11 ± 0.01 ^a^	-	-	-	-
C17:0	0.20 ± 0.00	-	-	-	-	-
C18:0	4.98 ± 0.14 ^b^	2.44 ± 0.29 ^a^	-	-	-	-
C18:1, cis-9	5.29 ± 0.22 ^a^	5.98 ± 0.61 ^b^	-	-	-	-
C18:2, cis-9,12	1.45 ± 0.11 ^a^	4.12 ± 0.61 ^b^	3.07	-	-	-
Saturated	87.73 ± 0.17 ^a^	91.65 ± 0.45 ^b^	48.65	31.27	40.15	32.11
Monounsaturated	5.84 ± 0.16 ^a^	6.09 ± 0.63 ^b^	47.65	43.31	50.76	45.76
Polyunsaturated	1.45 ± 0.11 ^a^	4.12 ± 0.05 ^b^	3.70	18.87	5.50	20.53

**Table 3:** Amino acid composition of beef and chicken *serunding* at week zero and week 24 of storage. ^abcd^ Values with different letters are significantly different across the same row (*p* < 0.05) within the same sample.

	Amino Acid (mg/g Protein)	Beef *Serunding*				Chicken *Serunding*			
		Week 0	Week 24			Week 0	Week 24		
		Control	25 ± 1 ⁰C	40 ± 1 ⁰C	60 ± 1 ⁰C	Control	25 ± 1 ⁰C	40 ± 1 ⁰C	60 ± 1 ⁰C
1	Glycine	12.69 ± 1.32 ^a^	12.57 ± 0.36 ^a^	10.94 ± 0.05 ^a^	11.86 ± 0.03 ^a^	9.95 ± 0.12 ^a^	9.52 ± 0.05 ^b^	8.74 ± 0.10 ^c^	9.61 ± 0.13 ^ab^
2	Histidine	12.97 ± 2.55 ^a^	7.18 ± 0.19 ^b^	6.58 ± 0.52 ^b^	6.38 ± 0.03 ^b^	6.75 ± 0.33 ^a^	6.16 ± 0.27 ^ab^	6.82 ± 0.11 ^a^	5.55 ± 0.14 ^b^
3	Arginine	27.64 ± 0.23 ^a^	21.88 ± 0.93 ^b^	18.01 ± 0.38 ^c^	16.70 ± 0.22 ^c^	23.46 ± 0.50 ^a^	20.68 ± 0.01 ^ab^	20.69 ± 0.27 ^ab^	19.97 ± 0.04 ^b^
4	Threonine	20.70 ± 0.98 ^a^	13.60 ± 0.36 ^b^	11.02 ± 0.16 ^c^	13.39 ± 0.09 ^b^	11.16 ± 1.22 ^a^	12.47 ± 0.17 ^a^	11.42 ± 0.09 ^a^	10.79 ± 0.01 ^a^
5	Alanine	21.33 ± 0.27 ^a^	19.09 ± 0.03 ^b^	15.71 ± 0.32 ^c^	15.61 ± 0.09 ^c^	23.28 ± 0.32 ^a^	20.65 ± 0.26 ^b^	20.10 ± 0.04 ^b^	14.38 ± 0.04 ^c^
6	Proline	11.05 ± 0.20 ^bc^	13.46 ± 0.51 ^a^	10.26 ± 0.24 ^c^	11.72 ± 0.11 ^b^	10.24 ± 0.47 ^a^	10.45 ± 0.11 ^a^	10.55 ± 0.17 ^a^	10.11 ± 0.0 ^a^
7	Tyrosine	17.18 ± 2.11 ^a^	11.49 ± 0.00 ^b^	8.87 ± 0.09 ^b^	11.51 ± 0.07 ^b^	10.62 ± 0.31 ^a^	9.09 ± 0.04 ^b^	8.01 ± 0.30 ^c^	8.61 ± 0.03 ^bc^
8	Methionine	9.95 ± 0.32 ^a^	6.27 ± 0.05 ^bc^	4.61 ± 0.46 ^c^	7.47 ± 0.64 ^b^	6.64 ± 0.01 ^a^	6.74 ± 0.25 ^b^	6.07 ± 0.01 ^b^	5.29 ± 0.35 ^b^
9	Isoleucine	13.75 ± 1.42 ^a^	13.75 ± 0.18 ^a^	10.29 ± 0.17 ^b^	12.68 ± 0.11 ^ab^	12.73 ± 0.26 ^a^	11.47 ± 0.17 ^b^	10.77 ± 0.39 ^b^	10.56 ± 0.00 ^b^
10	Leucine	16.36 ± 0.37 ^d^	25.40 ± 0.26 ^a^	18.72 ± 0.21 ^c^	21.69 ± 0.25 ^b^	19.35 ± 0.03 ^a^	17.32 ± 0.53 ^b^	18.14 ± 0.24a ^b^	17.33 ± 0.41 ^b^
11	Phenylalanine	119.59 ± 0.21 ^a^	114.27 ± 0.30 ^b^	63.50 ± 0.32 ^c^	61.80 ± 0.21 ^d^	98.55 ± 0.12 ^a^	98.37 ± 1.04 ^a^	85.10 ± 0.28 ^b^	83.27 ± 0.04 ^b^
12	Lysine	56.67 ± 0.43 ^a^	36.63 ± 0.24 ^b^	26.63 ± 0.96 ^c^	24.48 ± 0.36 ^d^	29.82 ± 0.09 ^a^	25.14 ± 0.71 ^b^	24.65 ± 0.11 ^b^	23.79 ± 0.14 ^b^
